# 3-Amino-*N*′-(2-oxoindolin-3-yl­idene)benzohydrazide

**DOI:** 10.1107/S1600536811029242

**Published:** 2011-07-30

**Authors:** Rifat Ara Jamal, Uzma Ashiq, Sammer Yousuf, Qurrat ul Ain

**Affiliations:** a Department of Chemistry, University of Karachi, Karachi 75270, Pakistan; b H.E.J. Research Institute of Chemistry, International Center for Chemical and Biological Sciences, University of Karachi, Karachi 75270, Pakistan

## Abstract

The title compound, C_15_H_12_N_4_O_2_, contains two substituted benzohydrazide and indole rings linked *via* a C=N double bond. The dihedral angle between the benzene ring and the indole ring system is 11.38 (10)°. The mol­ecular structure is stabilized by an intra­molecular N—H⋯O hydrogen bond, forming a six-membered ring. The crystal structure is consolidated by inter­molecular N—H⋯O and C—H⋯O inter­actions, which result in sheets.

## Related literature

For the biological activity of related compounds, see: Ashiq *et al.* (2008 ▶); Maqsood *et al.* (2006 ▶); Sarangapani & Reddy (1994 ▶). For related structures, see: Bai *et al.* (2006 ▶); Yang & Pan (2004 ▶).
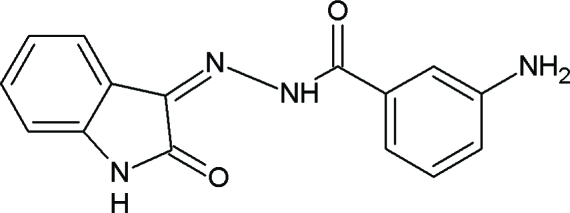


## Experimental

### 

#### Crystal data

C_15_H_12_N_4_O_2_
*M* *_r_* = 280.29
Monoclinic, 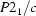

*a* = 8.8036 (8) Å
*b* = 8.9040 (7) Å
*c* = 17.0732 (14) Å
β = 92.335 (2)°
*V* = 1337.21 (19) Å^3^
*Z* = 4
Mo *K*α radiation
μ = 0.10 mm^−1^
*T* = 273 K
0.17 × 0.17 × 0.12 mm

#### Data collection

Bruker SMART APEX CCD area-detector diffractometer
Absorption correction: multi-scan (*SADABS*; Bruker, 2000 ▶) *T*_min_ = 0.984, *T*_max_ = 0.989
7637 measured reflections
2491 independent reflections
1551 reflections with *I* > 2σ(*I*)
*R*_int_ = 0.047

#### Refinement

*R*[*F* ^2^ > 2σ(*F* ^2^)] = 0.052
*wR*(*F* ^2^) = 0.122
*S* = 0.99
2491 reflections
198 parameters
1 restraint
H atoms treated by a mixture of independent and constrained refinement
Δρ_max_ = 0.18 e Å^−3^
Δρ_min_ = −0.17 e Å^−3^

### 

Data collection: *SMART* (Bruker, 2000 ▶); cell refinement: *SAINT* (Bruker, 2000 ▶); data reduction: *SAINT*; program(s) used to solve structure: *SHELXS97* (Sheldrick, 2008 ▶); program(s) used to refine structure: *SHELXL97* (Sheldrick, 2008 ▶); molecular graphics: *SHELXTL* (Sheldrick, 2008 ▶); software used to prepare material for publication: *SHELXTL*, *PARST* (Nardelli, 1995 ▶) and *PLATON* (Spek, 2009 ▶).

## Supplementary Material

Crystal structure: contains datablock(s) global, I. DOI: 10.1107/S1600536811029242/pv2431sup1.cif

Structure factors: contains datablock(s) I. DOI: 10.1107/S1600536811029242/pv2431Isup2.hkl

Supplementary material file. DOI: 10.1107/S1600536811029242/pv2431Isup3.cml

Additional supplementary materials:  crystallographic information; 3D view; checkCIF report

## Figures and Tables

**Table 1:** Hydrogen-bond geometry (Å, °)

*D*—H⋯*A*	*D*—H	H⋯*A*	*D*⋯*A*	*D*—H⋯*A*
N1—H1*A*⋯O2^i^	0.86	2.02	2.785 (2)	148
N3—H3*A*⋯O1	0.86	2.09	2.757 (2)	133
N4—H4*C*⋯N2^ii^	0.86 (2)	2.61 (2)	3.423 (3)	158 (2)
C12—H12*A*⋯O1^iii^	0.93	2.59	3.424 (3)	149

Symmetry codes: (i) 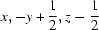; (ii) 
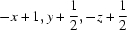
; (iii) 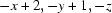.

**Fig. 1.**
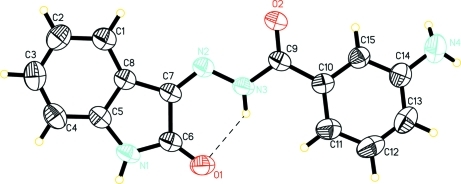
The molecular structure of the title molecule with displacement ellipsoids drawn at 50% probability level. The dashed lines indicate intramolecular hydrogen bond.

**Fig. 2.**
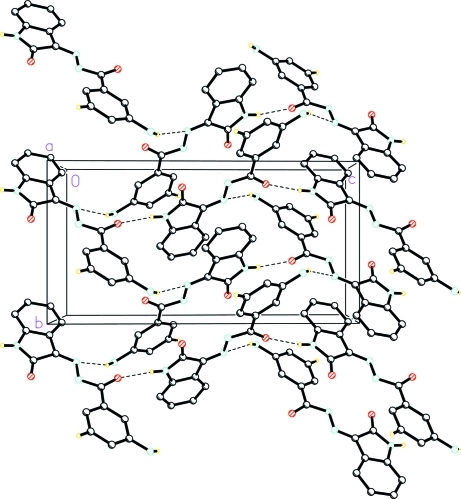
The crystal packing of the title compound.Hydrogen atoms not involved in hydrogen bonding have been excluded for clarity.

## supplementary crystallographic information

### Comment

Compounds containing hydrazide moiety have been revealed to exhibit a wide variety of interesting biological properties (Ashiq *et al.* 2008; Maqsood *et al.* 2006). In a quest to expand and further explore the biological significance of hydrazides, we have undertken a task to synthesize new Schiff bases of isatin with hydrazides. Isatins are very important compounds due to their antifungal properties (Sarangapani & Reddy, 1994). In this article we report the synthesis and crystal structure of the title compound.

The title molecule (Fig. 1) consists of substituted benzohydrazide and indole rings linked by C═N bond. The dihedral angle between the two substituted benzene (C10—C15) and indole ring (N1/C1—C8) is 11.38 (10)°. The bond lengths and angles are in normal range as in other structurally related compounds (Bai *et al.* 2006; Yang & Pan, 2004). The geometry of the molecule is stabilized by N3—H3A···O1 intramolecular hydrogen bond resulting in a six membered ring. In the crystal structure, the molecules are linked to form two-dimensional sheets *via* N1—H1A···O2, N4—H4C···N2 and C12—H12A···O1 intermolecular hydrogen bonds (Tab. 1 & Fig. 2).

### Experimental

To a solution of 2,3-indolinedione (10 mmol, 1.47 g) in 15 ml of ethanol with a few drops of glacial acetic acid and 3-aminobenzohydrazide (10 mmol, 1.51 g) in 15 ml ethanol were added. The mixture was refluxed for 2 h and a solid was obtained upon removal of the solvent by rotary evaporation. The resulting solid was washed with ethanol to afford the title compound (yield 75%). The crystals of the title compound suitable for XRD analysis were grown from a mixture of ethanol and methanol (1:1) by slow evaporation at room temperature.

### Refinement

H atoms on the C atoms and N1 and N3 were positioned geomatrically with C–H = 0.93 and N–H = 0.86 Å and constrained to ride on their parent atoms with *U*_iso_(H) = 1.2*U*_eq_(C/N). The H atoms on N4 were located from a difference Fourier map and refined isotropically.

### Crystal data

**Table:**

C_15_H_12_N_4_O_2_	*F*(000) = 584
*M**_r_* = 280.29	*D*_x_ = 1.392 Mg m^−^^3^
Monoclinic, *P*2_1_/*c*	Mo *K*α radiation, λ = 0.71073 Å
Hall symbol: -P 2ybc	Cell parameters from 1161 reflections
*a* = 8.8036 (8) Å	θ = 2.4–27.8°
*b* = 8.9040 (7) Å	µ = 0.10 mm^−^^1^
*c* = 17.0732 (14) Å	*T* = 273 K
β = 92.335 (2)°	Block, colorless
*V* = 1337.21 (19) Å^3^	0.17 × 0.17 × 0.12 mm
*Z* = 4	

### Data collection

**Table:**

Bruker SMART APEX CCD area-detector diffractometer	2491 independent reflections
Radiation source: fine-focus sealed tube	1551 reflections with *I* > 2σ(*I*)
graphite	*R*_int_ = 0.047
ω scans	θ_max_ = 25.5°, θ_min_ = 2.3°
Absorption correction: multi-scan (*SADABS*; Bruker, 2000)	*h* = −10→10
*T*_min_ = 0.984, *T*_max_ = 0.989	*k* = −10→10
7637 measured reflections	*l* = −20→20

### Refinement

**Table:**

Refinement on *F*^2^	Primary atom site location: structure-invariant direct methods
Least-squares matrix: full	Secondary atom site location: difference Fourier map
*R*[*F*^2^ > 2σ(*F*^2^)] = 0.052	Hydrogen site location: inferred from neighbouring sites
*wR*(*F*^2^) = 0.122	H atoms treated by a mixture of independent and constrained refinement
*S* = 0.99	*w* = 1/[σ^2^(*F*_o_^2^) + (0.0584*P*)^2^] where *P* = (*F*_o_^2^ + 2*F*_c_^2^)/3
2491 reflections	(Δ/σ)_max_ < 0.001
198 parameters	Δρ_max_ = 0.18 e Å^−^^3^
1 restraint	Δρ_min_ = −0.17 e Å^−^^3^

### Special details

**Table:**

Geometry. All e.s.d.'s (except the e.s.d. in the dihedral angle between two l.s. planes) are estimated using the full covariance matrix. The cell e.s.d.'s are taken into account individually in the estimation of e.s.d.'s in distances, angles and torsion angles; correlations between e.s.d.'s in cell parameters are only used when they are defined by crystal symmetry. An approximate (isotropic) treatment of cell e.s.d.'s is used for estimating e.s.d.'s involving l.s. planes.
Refinement. Refinement of *F*^2^ against ALL reflections. The weighted *R*-factor *wR* and goodness of fit *S* are based on *F*^2^, conventional *R*-factors *R* are based on *F*, with *F* set to zero for negative *F*^2^. The threshold expression of *F*^2^ > σ(*F*^2^) is used only for calculating *R*-factors(gt) *etc*. and is not relevant to the choice of reflections for refinement. *R*-factors based on *F*^2^ are statistically about twice as large as those based on *F*, and *R*- factors based on ALL data will be even larger.

### Fractional atomic coordinates and isotropic or equivalent isotropic displacement parameters (Å^2^)

**Table:**

	*x*	*y*	*z*	*U*_iso_*/*U*_eq_	
O1	0.66942 (18)	0.35101 (18)	−0.07613 (8)	0.0531 (5)	
O2	0.53184 (19)	0.37905 (18)	0.20442 (8)	0.0568 (5)	
N1	0.5172 (2)	0.1671 (2)	−0.13475 (10)	0.0470 (5)	
H1A	0.5578	0.1631	−0.1797	0.056*	
N2	0.4730 (2)	0.27880 (19)	0.06115 (9)	0.0407 (5)	
N3	0.5848 (2)	0.38136 (19)	0.07676 (9)	0.0415 (5)	
H3A	0.6377	0.4169	0.0398	0.050*	
N4	0.8354 (3)	0.8078 (3)	0.32398 (15)	0.0676 (7)	
C1	0.2412 (3)	0.0351 (3)	−0.00233 (13)	0.0488 (6)	
H1B	0.2175	0.0545	0.0493	0.059*	
C2	0.1601 (3)	−0.0703 (3)	−0.04635 (15)	0.0557 (7)	
H2A	0.0815	−0.1229	−0.0241	0.067*	
C3	0.1950 (3)	−0.0980 (3)	−0.12350 (14)	0.0569 (7)	
H3B	0.1379	−0.1682	−0.1524	0.068*	
C4	0.3119 (3)	−0.0245 (3)	−0.15845 (13)	0.0523 (7)	
H4A	0.3354	−0.0442	−0.2101	0.063*	
C5	0.3923 (2)	0.0790 (2)	−0.11405 (12)	0.0417 (6)	
C6	0.5659 (3)	0.2585 (3)	−0.07583 (12)	0.0409 (6)	
C7	0.4654 (2)	0.2238 (2)	−0.00862 (11)	0.0374 (5)	
C8	0.3583 (2)	0.1110 (2)	−0.03664 (12)	0.0391 (5)	
C9	0.6110 (2)	0.4264 (2)	0.15255 (12)	0.0406 (6)	
C10	0.7362 (2)	0.5353 (2)	0.16743 (12)	0.0400 (6)	
C11	0.8540 (3)	0.5576 (3)	0.11661 (13)	0.0518 (6)	
H11A	0.8582	0.5027	0.0704	0.062*	
C12	0.9645 (3)	0.6629 (3)	0.13619 (15)	0.0606 (7)	
H12A	1.0442	0.6778	0.1030	0.073*	
C13	0.9587 (3)	0.7457 (3)	0.20365 (16)	0.0586 (7)	
H13A	1.0342	0.8161	0.2154	0.070*	
C14	0.8416 (3)	0.7259 (3)	0.25467 (14)	0.0490 (6)	
C15	0.7329 (3)	0.6181 (2)	0.23602 (13)	0.0440 (6)	
H15A	0.6555	0.6009	0.2704	0.053*	
H4C	0.7478 (19)	0.819 (3)	0.3435 (17)	0.099 (12)*	
H4B	0.889 (4)	0.894 (3)	0.3239 (17)	0.101 (11)*	

### Atomic displacement parameters (Å^2^)

**Table:**

	*U*^11^	*U*^22^	*U*^33^	*U*^12^	*U*^13^	*U*^23^
O1	0.0485 (10)	0.0635 (11)	0.0480 (9)	−0.0019 (9)	0.0114 (8)	0.0040 (8)
O2	0.0650 (11)	0.0715 (12)	0.0346 (8)	−0.0188 (9)	0.0111 (8)	−0.0022 (8)
N1	0.0485 (12)	0.0630 (12)	0.0299 (9)	0.0067 (11)	0.0055 (9)	0.0003 (9)
N2	0.0402 (11)	0.0452 (11)	0.0366 (10)	0.0030 (9)	0.0024 (9)	0.0015 (8)
N3	0.0439 (11)	0.0476 (11)	0.0334 (10)	−0.0005 (10)	0.0080 (9)	0.0016 (8)
N4	0.0598 (18)	0.0655 (16)	0.0765 (16)	−0.0067 (14)	−0.0089 (14)	−0.0142 (14)
C1	0.0431 (14)	0.0557 (15)	0.0473 (13)	0.0064 (13)	0.0012 (12)	0.0011 (12)
C2	0.0413 (15)	0.0549 (16)	0.0704 (17)	0.0005 (13)	−0.0028 (13)	0.0059 (14)
C3	0.0515 (16)	0.0529 (15)	0.0644 (17)	0.0076 (13)	−0.0192 (14)	−0.0053 (13)
C4	0.0566 (17)	0.0566 (15)	0.0429 (13)	0.0126 (14)	−0.0088 (13)	−0.0042 (12)
C5	0.0392 (13)	0.0476 (13)	0.0377 (12)	0.0125 (12)	−0.0042 (11)	0.0000 (11)
C6	0.0366 (14)	0.0488 (14)	0.0372 (12)	0.0113 (12)	0.0017 (11)	0.0042 (11)
C7	0.0364 (13)	0.0432 (13)	0.0326 (11)	0.0115 (11)	0.0013 (10)	0.0028 (10)
C8	0.0354 (12)	0.0437 (13)	0.0379 (11)	0.0089 (11)	−0.0004 (10)	0.0025 (10)
C9	0.0436 (14)	0.0440 (13)	0.0345 (12)	0.0075 (11)	0.0040 (11)	0.0023 (10)
C10	0.0361 (13)	0.0412 (13)	0.0426 (12)	0.0055 (11)	0.0021 (10)	0.0070 (11)
C11	0.0452 (15)	0.0603 (16)	0.0504 (13)	0.0042 (13)	0.0079 (12)	0.0053 (12)
C12	0.0403 (15)	0.0737 (18)	0.0685 (17)	−0.0012 (14)	0.0084 (14)	0.0180 (15)
C13	0.0447 (16)	0.0558 (16)	0.0743 (17)	−0.0074 (13)	−0.0084 (14)	0.0100 (14)
C14	0.0428 (15)	0.0479 (14)	0.0557 (14)	0.0047 (12)	−0.0055 (13)	0.0030 (12)
C15	0.0377 (13)	0.0472 (14)	0.0470 (13)	0.0014 (11)	−0.0012 (11)	0.0047 (11)

### Geometric parameters (Å, °)

**Table:**

O1—C6	1.228 (2)	C3—H3B	0.9300
O2—C9	1.224 (2)	C4—C5	1.372 (3)
N1—C6	1.351 (3)	C4—H4A	0.9300
N1—C5	1.407 (3)	C5—C8	1.396 (3)
N1—H1A	0.8600	C6—C7	1.509 (3)
N2—C7	1.287 (2)	C7—C8	1.446 (3)
N2—N3	1.361 (2)	C9—C10	1.482 (3)
N3—C9	1.365 (2)	C10—C15	1.385 (3)
N3—H3A	0.8600	C10—C11	1.393 (3)
N4—C14	1.393 (3)	C11—C12	1.382 (3)
N4—H4C	0.858 (10)	C11—H11A	0.9300
N4—H4B	0.90 (3)	C12—C13	1.370 (3)
C1—C2	1.382 (3)	C12—H12A	0.9300
C1—C8	1.383 (3)	C13—C14	1.388 (3)
C1—H1B	0.9300	C13—H13A	0.9300
C2—C3	1.387 (3)	C14—C15	1.383 (3)
C2—H2A	0.9300	C15—H15A	0.9300
C3—C4	1.376 (3)		
			
C6—N1—C5	112.13 (18)	N2—C7—C8	125.39 (19)
C6—N1—H1A	123.9	N2—C7—C6	128.0 (2)
C5—N1—H1A	123.9	C8—C7—C6	106.59 (17)
C7—N2—N3	116.52 (17)	C1—C8—C5	119.6 (2)
N2—N3—C9	118.39 (17)	C1—C8—C7	133.39 (19)
N2—N3—H3A	120.8	C5—C8—C7	107.01 (18)
C9—N3—H3A	120.8	O2—C9—N3	120.4 (2)
C14—N4—H4C	117 (2)	O2—C9—C10	122.86 (19)
C14—N4—H4B	113.9 (19)	N3—C9—C10	116.76 (18)
H4C—N4—H4B	113 (3)	C15—C10—C11	119.5 (2)
C2—C1—C8	118.6 (2)	C15—C10—C9	116.88 (19)
C2—C1—H1B	120.7	C11—C10—C9	123.6 (2)
C8—C1—H1B	120.7	C12—C11—C10	118.7 (2)
C1—C2—C3	120.5 (2)	C12—C11—H11A	120.6
C1—C2—H2A	119.7	C10—C11—H11A	120.6
C3—C2—H2A	119.7	C13—C12—C11	121.2 (2)
C4—C3—C2	121.7 (2)	C13—C12—H12A	119.4
C4—C3—H3B	119.2	C11—C12—H12A	119.4
C2—C3—H3B	119.2	C12—C13—C14	120.9 (2)
C5—C4—C3	117.3 (2)	C12—C13—H13A	119.6
C5—C4—H4A	121.4	C14—C13—H13A	119.6
C3—C4—H4A	121.4	C15—C14—C13	117.9 (2)
C4—C5—C8	122.3 (2)	C15—C14—N4	120.5 (2)
C4—C5—N1	128.8 (2)	C13—C14—N4	121.5 (2)
C8—C5—N1	108.91 (19)	C14—C15—C10	121.7 (2)
O1—C6—N1	127.9 (2)	C14—C15—H15A	119.1
O1—C6—C7	126.8 (2)	C10—C15—H15A	119.1
N1—C6—C7	105.3 (2)		
			
C7—N2—N3—C9	−170.74 (18)	N1—C5—C8—C7	0.4 (2)
C8—C1—C2—C3	−0.5 (3)	N2—C7—C8—C1	0.8 (4)
C1—C2—C3—C4	1.1 (4)	C6—C7—C8—C1	179.3 (2)
C2—C3—C4—C5	−0.6 (3)	N2—C7—C8—C5	−177.82 (19)
C3—C4—C5—C8	−0.4 (3)	C6—C7—C8—C5	0.6 (2)
C3—C4—C5—N1	178.9 (2)	N2—N3—C9—O2	−2.5 (3)
C6—N1—C5—C4	179.2 (2)	N2—N3—C9—C10	178.53 (17)
C6—N1—C5—C8	−1.4 (2)	O2—C9—C10—C15	−19.6 (3)
C5—N1—C6—O1	−178.1 (2)	N3—C9—C10—C15	159.37 (19)
C5—N1—C6—C7	1.7 (2)	O2—C9—C10—C11	160.6 (2)
N3—N2—C7—C8	178.30 (18)	N3—C9—C10—C11	−20.5 (3)
N3—N2—C7—C6	0.2 (3)	C15—C10—C11—C12	−0.2 (3)
O1—C6—C7—N2	−3.2 (4)	C9—C10—C11—C12	179.6 (2)
N1—C6—C7—N2	177.0 (2)	C10—C11—C12—C13	−0.7 (4)
O1—C6—C7—C8	178.4 (2)	C11—C12—C13—C14	0.2 (4)
N1—C6—C7—C8	−1.4 (2)	C12—C13—C14—C15	1.2 (4)
C2—C1—C8—C5	−0.5 (3)	C12—C13—C14—N4	179.1 (2)
C2—C1—C8—C7	−179.0 (2)	C13—C14—C15—C10	−2.2 (3)
C4—C5—C8—C1	1.0 (3)	N4—C14—C15—C10	179.9 (2)
N1—C5—C8—C1	−178.47 (19)	C11—C10—C15—C14	1.7 (3)
C4—C5—C8—C7	179.85 (19)	C9—C10—C15—C14	−178.15 (19)

### Hydrogen-bond geometry (Å, °)

**Table:**

*D*—H···*A*	*D*—H	H···*A*	*D*···*A*	*D*—H···*A*
N1—H1A···O2^i^	0.86	2.02	2.785 (2)	148
N3—H3A···O1	0.86	2.09	2.757 (2)	133
N4—H4C···N2^ii^	0.86 (2)	2.61 (2)	3.423 (3)	158 (2)
C12—H12A···O1^iii^	0.93	2.59	3.424 (3)	149

Symmetry codes: (i) *x*, −*y*+1/2, *z*−1/2; (ii) −*x*+1, *y*+1/2, −*z*+1/2; (iii) −*x*+2, −*y*+1, −*z*.
